# Editorial and Miscellaneous

**Published:** 1866-10

**Authors:** 


At the recent Annual Commencement of Atlanta Medical College, the following Address to the Faculty, which had been previously prepared in accordance with a resolution adopted at a meeting of the Graduating Class, was read by A. H. Brantley:

Gentlemen:

Profoundly grateful for the cordial kindness and deep interest which you have, through all the onerous duties of the present session, so continuously exercised towards each and every one of us, we, as a Class, are desirous that no opportunity shall escape us of assuring you of our highest esteem and appreciation.

In taking leave of you, we feel that we are going away from those who have been our friends indeed, and your memories we take with us as precious jewels saved from the gulf that *must* soon claim to-day.

This continued kindness on your part has filled our hearts with grateful feelings of respect and esteem that *must* defy the corroding hand of time.

You have led us to the threshold of the grand Temple of Science, and with feelings of awe and thrilling wonder we gaze upon its stupendous proportions. You have pointed out to us the vast, the illimitable field that lies before us, and with hopeful, cheering words you send us to the harvest. If our labors are crowned with honorable success, the laurels belong not to us, but to *you*.

Our Alma Mater buckles on our armor and bids us God-speed in the struggle of Life, and for her sake, we, this day, pledge ourselves, and the best energies of our lives, to bear aloft, for the cause of humanity, the proud Banner of Science which she has placed in our hands.

We trust that we are fully alive to the exigencies of the new era that is dawning, and that we properly appreciate the heavy responsibilities devolving upon us.

That there are difficulties before us, we know. Mountains, dark, and rough, and rugged, that have daunted many a brave spirit, and defied the accumulated wisdom of ages, lift their wild, craggy cliffs before us, and cast their gloomy shadows athwart our pathway, but by the clear light of those truths which you have so faithfully, and at the cost of so much valuable time and labor, endeavored to inculcate in our minds, we hope to pass those dizzy hights and open up the way to *greater* and *grander* results that *must, ere long*, burst with their wonderful brilliancy upon the scientific world.

In parting with you, we earnestly beg to *assure* you that nothing exists in the heart of *any* student of this Class that can ever mar the memory of our relations as teachers and students. Long years from to-day, when our brows are marred by the ravages of time, and some of us are sleeping away back, among the green hills of the past, our hearts will swell with emotion as we gaze on these sacred walls through the mists of those weary years, and we will murmur then as we murmur now: "May God bless the Faculty of the Atlanta Medical College!"

MEDICAL CLASS.

*Atlanta, Ga.*, August 28th, 1866.

J. A. HUNNICUTT, Chairman of Meeting.
J. L. M. HARDMAN, Secr'y.

A. H. Brantley, (Chm'n,) Julius C. Sosnowski, S. S. Smithwick, Wm. O'Daniel and M. Edwards, *Committee.*

---

In response, Professor Alexander Means made some pathetic remarks, and afterwards read the following original lines:

On this bright gala-day, busy memory sweeps
Upon broad dusky wing; the exuberant past
Numbers twenty-three months, and looks down on the heaps
Of a war-ravaged city, just breathing her last.
O God! what a vision glares red on the eye,
As earth-rocking thunders roll death through the streets;
And millions of capital melt in the sky
As flames lash her buildings in wild, livid sheets.
Pandemonium shouts through her sulphurous hall,
'Till the revel, infernal, re-echos through Hell;
And the great Master Spirit, responds to the call,
That invokes his black curse over mountain and dell.
But enough—there's a chapter of carnage and blood,
That shall glow in red letters on history's page,
And shall rival the records of fire and flood,
That have scandal'd a Nero's and Attila's age.
The Demon of War had scarce quitted his prey,
And a conquering army its plunder and lust,
Its cataract roar had but just died away
Over bomb-shatter'd buildings, now crumbled to dust,
When thousands who fled from their blazing abodes,
To seek among strangers, a covert from war,
Look'd longingly back o'er the blood-clotted roads,
And their courage re-plum'd under hope's guiding star.
From the North, South and East, the worn refugees come,
And the West pours her quota of dust-cover'd throngs;
Each sweeps o'er the ruins of his once happy home,
And appeals to high Heaven to avenge all his wrongs.
Full-soul'd and harmonious, they rush to new toils
And tax earth and air, sea and sky, for supplies;
And though myrmidon legions had gorged on her spoils,
They swear by their manhood, "Atlanta shall rise."
'Twas a struggle of giants that knew no recoil—
From morning till midnight resounded their blows- –
The ingathering thousands no dangers could foil,
Till the white-flag of triumph in glory arose.
Old Balbec and Luxor for ages have slept,
Redeemless and time-worn, and shrouded in gloom;
O'er their huge broken columns the serpent has crept,
And the yells of the jackall have sounded their doom.
But the deathless "Gate City," though crushed by the tread
Of militant millions and thundering trains,
Has rent her own winding-sheet, burst from the dead,
And the new pulse of life gushes warm through her veins.

Hail! hail! ye proud piles of undying renown,
Your numbers shall swell as the ages roll on;
And your sun-lighted summits in grandeur look down
On the penitent gazers thy fame shall have won.
Fair Queen of the mid-lands, thy reign shall extend
From mountain to sea-board, where commerce is found;
And religion and science in harmony blend,
To foster the virtues their bulwarks surround.
Thus lighting the landscape and blessing the lands,
The next generation thy name shall inspire,
To shout on the soil where thy monument stands,
That the *souls* of thy people were proof against fire.
Ye parchmented heirs of Hippocrates, rouse,
Your knowledge must quacks and imposters confound;
Your fond Alma Mater has laurel'd your brows,
And Atlanta shall honor the sons she has crown'd.
She has own'd your profession—its temple repaired—
Dismantled and torn by the storm that has pass'd;
Then build up her fame, with a labor unspar'd,
Till her glory eclipses the gloom of the past.

